# Supplementary material for: The earliest dipodomyine heteromyid in North America and the phylogenetic relationships of geomorph rodents
Source: PeerJ. 2023 Mar 8;11:e14693. doi: 10.7717/peerj.14693 (PMC10007967; doi:10.7717/peerj.14693)
Supplement: Table S1 [file peerj-11-14693-s001.docx]

**Supplemental Table S1.** Modern and fossil specimens included in the phylogenetic analyses of this study.

| **Genus** | **Species** | **Specimens** |
| --- | --- | --- |
| **Outgroup - Eomyidae** | |  |
| *Paradjidaumo* | *trilophus* | LACM CIT 618, LACM 4896 |
| **Florentiamyidae** |  |  |
| *Florentiamys* | *kingi* | AMNH 103381, 103384 |
| *Hitonkala* | *andersontau* | SDSM 56120, LACM 9268, AMNH 128986 |
| *Sanctimus* | *simonisi* | AMNH 103380 |
| *Sanctimus* | *stouti* | AMNH 103385 |
| *Sanctimus* | *stuartae* | LACM 15292 |
| **Geomyoidea incertae sedis** | |  |
| *Balantiomys* | *oregonensis* | LACM CIT 370-373, 4001 |
| *Harrymys* | *irvini* | UCMP 122004 |
| *Mioheteromys* | *amplissimus* | UF 170551 (cast) |
| *Mojavemys* | *galushai* | AMNH 129676; USNM V49854 |
| *Phelosaccomys* | *neomexicanus* | AMNH 129620, USNM V49855 |
| *Proharrymys* | *schlaikjeri* | LACM 9326, 15291 |
| *Proheteromys* | *latidens* | UCMP 82276, 82335, 150688 |
| *Tenudomys* | *dakotensis* | LACM 17038; SDSM 54248 |
| *Trogomys* | *rupinimenthae* | LACM CIT 5184-5190 |
| **Heteromyidae - Dipodomyinae** | |  |
| *Cupidinimus* | *nebraskensis* | CM 10173, 10175, 10193; USNM PAL352, V21530 |
| *Dipodomys* | *merriami* | CM 5039, 5042, 5062, 5064, 5106, 6253, 6254,  6330, 6405, 6417, 6474, 6519, 6606, USNM 32772,  54552, 108884, 203017 |
| *Microdipodops* | *megacephalus* | USNM 24417, 31823, 68081, 78216, 78217, 80128,  101226, 101227, 101228, 101229, 101230, 244584,  244586, 246039 |
| *Prodipodomys* | sp. | AMNH F:AM 87427 |
| **Heteromyidae - Heteromyinae** | |  |
| *Heteromys* | *desmarestianus* | CM 91951, 118618, 118619, 118620, 118621,  118622, 118634, 118628, 118632, 118635, 118639,  USNM 63719, 170976, 171107, 179016, 250348 |
| *Heteromys* | *pictus* | CM 13700, 13701, 103541, 103542, 103543,  103555, 103556, 103557, 103560, 103564,  103465, 103566, 103575, USNM 71510 |
| **Heteromyidae - Perognathinae** | |  |
| *Bursagnathus* | *aterosseus* | UCMP 56279, JODA 7407 |
| *Chaetodipus* | *artus* | MVZ 85677, USNM 96298 |
| *Chaetodipus* | *hispidus* | CM 16235, 16298, 48699, 48701, 48702,48704,  65332, 65333, 65334, 65337, 65338, 89207, 91055,  108273, USNM 348445 |
| *Mioperognathus* | *willardi* | AMNH 129674 |
| *Perognathus* | *furlongi* | LACM CIT 35 |
| *Perognathus* | *amplus* | USNM 46711, 212780 |
| **Heteromyidae** |  |  |
| *Schizodontomys* | *amnicolus* | AMNH 129659, UNSM 26682, 26685-26686 |
| *Schizodontomys* | *harkseni* | LACM 5510-5512, 9257 |
| *Schizodontomys* | *sulcidens* | AMNH 13758, 103884, 129579, 129618 |
| **Geomyidae - Entoptychinae** | |  |
| *Entoptychus* | sp. | UCMP 65251 |
| *Gregorymys* | *formosus* | AMNH 12887; SDSM 6257, 6297 |
| *Pleurolicus* | *sulcifrons* | AMNH 7175, 7180, 7181, 7182, 7185,  UCMP 69425, 86191, 86196, 86198,  UWBM 73449, 73465 |
| **Geomyidae - Geomyinae** | |  |
| *Cratogeomys* | *merriami* | USNM 5498, 53494, 54304, 54306, 57963, 57964,  58167, 58169, 58170, 58171, 58172, 50109, 50110,  53497, 54299, 54300, 54307, 54308, 55346, 55347,  55348, 57970, 58166, 58168 |
| *Geomys* | *arenarius* | AMNH 132030, CM 90965, 90966, 90967,  90968, 90969, 90970, 90971, 90972, USNM 18012,  18117, 20314, 20315, 35599, 58330, 58332 |
| *Parapliosaccomys* | cf. *P. oregonensis* | LACM 156176-156182, 156184, 156185, 156201,  156206, 156212, 156218, 156222, 156226, 156227,  156242, 156245; SDSM 59863-59864, 59842 |
| *Pliosaccomys* | *dubius* | LACM CIT 1796-1800, 1805, 1807-1808 |
| *Thomomys* | *talpoides* | CM 298, 717, 720, 765, 2615, 5592, 5621, 11861,  11862, 11865, 15447, 21494, 21496, 24267, 28781,  67096, 91066, 91073, 150725, 158072, 158079,  213345, 213614, 213826, 242445, 248222 |
