# Supplementary material for: The earliest dipodomyine heteromyid in North America and the phylogenetic relationships of geomorph rodents
Source: PeerJ. 2023 Mar 8;11:e14693. doi: 10.7717/peerj.14693 (PMC10007967; doi:10.7717/peerj.14693)
Supplement: Table S2 — Dental measurements follow Carrasco (2000), cranial measurements follow Korth & Samuels (2015), and postcranial measurements follow Samuels & Van Valkenburgh (2008). [file peerj-11-14693-s002.docx]

**Supplemental Table S2.** Definitions of cranial, dental, and postcranial measurements included in this study. Dental measurements follow Carrasco (2000), cranial measurements follow Korth & Samuels (2015), and postcranial measurements follow Samuels & Van Valkenburgh (2008).

| **Abbreviation** | **Definition** |
| --- | --- |
| SkullL | Skull length measured ventrally from occipital condyle to anterior margin of incisor |
| MCW | Maximum cranial width |
| OrbW | Interorbital width |
| NasalL | Nasal length |
| RostW | Width of rostrum at posterior margin |
| RostD | Depth of rostrum at posterior margin |
| BullaL | Auditory bulla length |
| BullaW | Auditory bulla width |
| RPL | Rostral perforation length |
| RPD | Rostral perforation depth |
| UDiastL | Upper diastema length |
| ForMagW | Foramen magnum width |
| DentL | Dentary length measured from condyle to tip of incisor |
| LDiastL | Lower diastema length |
| dentDm1 | Depth of dentary measured below m1 anterior root |
| I1L | Upper incisor anteroposterior length measured at base of blade |
| I1W | Upper incisor mediolateral width measured at base of blade |
| P4L | Length of P4 measured at occlusal surface |
| P4W | Width of P4 measured at occlusal surface |
| M1L | Length of M1 measured at occlusal surface |
| M1W | Width of M1 measured at occlusal surface |
| M2L | Length of M2 measured at occlusal surface |
| M2W | Width of M2 measured at occlusal surface |
| M3L | Length of M3 measured at occlusal surface |
| M3W | Width of M3 measured at occlusal surface |
| P4-M3L | Upper toothrow length measured at alveolar level |
| i1L | Lower incisor anteroposterior length measured at base of blade |
| i1W | Lower incisor mediolateral width measured at base of blade |
| p4L | Length of p4 measured at occlusal surface |
| p4W | Width of p4 measured at occlusal surface |
| m1L | Length of m1 measured at occlusal surface |
| m1W | Width of m1 measured at occlusal surface |
| m2L | Length of m2 measured at occlusal surface |
| m2W | Width of m2 measured at occlusal surface |
| m3L | Length of m3 measured at occlusal surface |
| m3W | Width of m4 measured at occlusal surface |
| p4-m3L | Lower toothrow length measured at alveolar level |
| mt3W | Mediolateral diameter of the 3rd metatarsal measured midshaft |
| mt4W | Mediolateral diameter of the 4th metatarsal measured midshaft |
| pph3L | Length of the proximal phalanx of pes digit 3 |
| pph3W | Mediolateral diameter of the proximal phalanx of digit 3 measured midshaft |
| pph4L | Length of the proximal phalanx of pes digit 4 |
| pph4W | Mediolateral diameter of the proximal phalanx of digit 4 measured midshaft |
