# Supplementary material for: The earliest dipodomyine heteromyid in North America and the phylogenetic relationships of geomorph rodents
Source: PeerJ. 2023 Mar 8;11:e14693. doi: 10.7717/peerj.14693 (PMC10007967; doi:10.7717/peerj.14693)
Supplement: Table S4 — A complete matrix of character states for all studied taxa is provided in Table S5, while data files used in parsimony and Bayesian analyses are provided in Data S1 and S2, respectively. [file peerj-11-14693-s004.docx]

**Supplemental Table S4.** Character list and character states for phylogenetic analysis. A complete matrix of character states for all studied taxa is provided in Table S5, while data files used in parsimony and Bayesian analyses are provided in Data S1 and S2, respectively.

1. Anterior alar fissure rises: (0) far posterior to M3, (1) just posteriorly to M3, (2) above or anterior to M3. [ordered]
2. Anterior edge of anterior alar fissure: (0) alisphenoid only, (1) palatine and alisphenoid
3. Posterior alar fissure: (0) absent, (1) present but separated from foramen ovale, (2) present and joined with foramen ovale
4. Suture of maxilla and alisphenoid (lateral view): (0) none, (1) narrow, (2) broad. [ordered]
5. Dorsal extent of alisphenoid: (0) low, (1) moderate, (2) high. [ordered]
6. Anterior division of temporalis arising on alisphenoid: (0) absent, (1) present.
7. Bone of the bulla: (0) single lamina, (1) trabeculate.
8. Anterior inflation of bulla: (0) absent, (1) present, (2) meeting at midline. [ordered]
9. Ventral inflation of auditory bulla: (0) absent, (1) small, (2) large. [ordered]
10. Lateral inflation of bulla: (0) absent, (1) small, (2) large. [ordered]
11. Posterior inflation of mastoid: (0) absent, (1) small, (2) large. [ordered]
12. Dorsal inflation of mastoid: (0) absent, (1) small, (2) great, (3) joining bulla anterior to meatus. [ordered]
13. Lateral inflation of mastoid: (0) absent, (1) small, (2) large. [ordered]
14. Anteromedial bullar processes: (0) absent, (1) present, (2) present and meeting in midline. [ordered]
15. Mastoid foramen: (0) absent, (1) minute, (2) large
16. Angle of the mandible: (0) straight, (1) deflected.
17. Angle of mandible: (0) large, (1) reduced
18. Relative height of coronoid process: (0) tall, (1) reduced, (2) very reduced. [ordered]
19. Insertion of masseter on dentary: (0) smooth or nearly smooth, (1) marked by a strong anterior ridge
20. Anterior end of masseteric fossa: (0) posterior to p4, (1) ventral to p4, (2) anterior to p4. [ordered]
21. Mental foramen: (0) anteroventral to masseter insertion, (1) anterodorsal to masseter insertion
22. Posterior end of incisor alveolus: (0) in plane of mandible, (1) projects laterally from mandible
23. Buccinator and masticatory foramina: (0) separate, (1) fused.
24. Accessory foramen ovale on alisphenoid: (0) present and complete, (1) present but lacking posterior margin, (2) absent.
25. Masticatory and buccinators foramina: (0) separated from accessory foramen ovale, (1) united with accessory foramen ovale
26. Foramen ovale: (0) surrounded by alisphenoid bone, (1) bounded posteriorly by auditory bulla
27. Central groove on upper incisor: (0) absent, (1) present.
28. Distinct tubercle or swelling at posteroventral border of infraorbital foramen: (0) absent, (1) small, (2) large. [ordered]
29. Infraorbital canal low: (0) lateral to rostrum, (1) depressed into rostrum, (2) depressed but laterally bulging
30. Shape of interparietal bone: (0) triangular, (1) oval
31. Interparietal: (0) without bullar constriction, (1) with some constriction, (2) with great constriction. [ordered]
32. Interparietal: (0) wide or constricted by bullar inflation, (1) narrow but not constricted by bullar inflation
33. Supraorbital bony flange: (0) absent, (1) present
34. Optic foramen separation from orbital fissure: (0) wide, (1) narrow, (2) absent
35. Size of optic foramen: (0) <1mm in diameter, (1) >1mm in diameter.
36. Ethmoid foramen dorsal to: (0) M3, (1) mid M2 to M2/M3 junction, (2) mid M2 to mid M1 [ordered]
37. Sphenofrontal foramen: (0) present, (1) absent
38. Unossified area on between maxillary and lacrimal bones: (0) absent, (1) present
39. Unossified area dorsal to orbitosphenoid: (0) absent, (1) present
40. Parapterygoid fossae: (0) absent, (1) shallow, (2) deep, (3) very deep.
41. Sphenopterygoid canal: (0) absent, (1) present
42. Posterior maxillary notch: (0) open, (1) closed, (2) absent.
43. Postglenoid foramen: (0) present, (1) absent
44. Postglenoid foramen: (0) in squamosal bone, (1) in squamosal bone/another between squamosal and periotic, (2) between bones and continuous with posterior alar fissure
45. Hamular process of pterygoid robust (0); or thin (1).
46. Incisive foramen length/diastemal length: (0) >0.40, (1) 0.20 to 0.40, (2) 0.10 to 0.19. [ordered]
47. Location of the premaxillary-maxillary suture crossing of the midline of the palate: (0) at the posterior end of the incisive foramen, (1) 1/3rd from posterior margin of the incisive foramen, (2) halfway through the incisive foramen. [ordered]
48. Rostrum: (0) not tapered, (1) tapered by descending nasals, (2) elevated nasals
49. Masseter: (0) protrogomorphous, (1) sciuromorphous
50. Lateral wall of rostrum: (0) solid, (1) perforated at and anterior to infraorbital foramen
51. Interpremaxillary foramen: (0) absent, (1) present
52. Posterior end of nasals: (0) extending farther posteriorly than premaxillae, (1) aligned with posterior end of premaxillae, (2) anteriorly retracted, (3) surrounded posteriorly by premaxillae. [ordered]
53. Squamosal: (0) not reduced, (1) reduced to thin bar posteriorly, (2) overcome by mastoid and bulla. [O]
54. Squamosal: (0) entire posteriorly, (1) emarginate posteriorly dorsal to auditory bulla.
55. Boss anterior to glenoid fossa that redirects temporal muscle: (0) absent, (1) small, (2) very prominent. [ordered]
56. Anterior squamosal foramen: (0) absent, (1) present.
57. Stapedial foramen: (0) present, (1) absent.
58. Stapedial canal: (0) open, (1) enclosed by bone
59. Upper tooth rows: (0) parallel, (1) posteriorly diverging
60. Anterior surface of the lower incisors: (0) convex, (1) flat.
61. Temporal foramen: (0) present, (1) absent.
62. Length of scar for temporalis muscle: (0) complete to occipital, (1) shortened.
63. Parietal: (0) reaches occiput, (1) somewhat retreated from occiput, (2) does not come near occiput. [ordered]
64. Origins of temporal muscles: (0) reach or come close to midline, (1) restricted laterally, (2) restricted very far laterally. [ordered]
65. Posterior margin of anterior root of zygomatic arch: (0) lateral to P4, (1) slightly anterior to P4, (2) far anterior to P4.
66. Vacuity anterior to bulla: (0) absent, (1) present
67. Sphenopalatine foramen: (0) dorsal to M2 or M2-M3 boundary, (1) dorsal to M1 or M1-M2 boundary, (2) dorsal to P4 or P4-M1 boundary
68. Posterior palatine foramina: (0) within palatine, (1) within palatine-maxillary suture
69. Furrows of the palate: (0) absent, (1) shallow, (2) deep.
70. Foramen magnum: (0) posteriorly oriented, (1) posteroventrally oriented/anteriorly shifted
71. Space between auditory bullae and basioccipital: (0) absent, (1) present
72. Fissure medial to bulla: (0) absent, (1) present
73. Component of the basicranium continuous between basioccipital and basisphenoid: (0) not swollen, (1) swollen
74. Mental foramen: (0) near posterior end of diastema, (1) near midpoint of diastema
75. Foramen between m3 and coronoid process: (0) absent, (1) present
76. Posterior process of coronoid: (0) absent, (1) present
77. Mandibular condyle shape: (0) anteroposteriorly oriented, (1) transversely expanded
78. Premaxilla-frontal suture dorsally: (0) simple, (1) interdigitized.
79. Jugal contribution to glenoid fossa: (0) absent, (1) forming lateral wall of the fossa, (2) forming anterolateral corner of the fossa.
80. Anterior projection of frontal between premaxilla and jugal: (0) absent, (1) present
81. Glenoid fossa of the squamosal: (0) anterodorsal to the auditory region, (1) near the auditory region.
82. Hamulus-bulla contact: (0) present, (1) absent.
83. Nasals: (0) retracted posterior to or near level with incisors and premaxilla, (1) expanded far anteriorly of premaxilla and incisors.
84. Posterior border of anterior root of zygomatic arch: (0) at the level of the posterior end of the nasals and/or premaxillae, (1) posterior to the posterior end of the nasals and/or premaxillae, (2) anterior to the posterior end of the nasals and/or premaxillae.
85. P4: (0) smaller than the molars or (1) larger than the molars.
86. Chevrons: (0) absent or (1) present.
87. Protostyle of P4: (0) absent or (1) present.
88. Entostyle and/or protostyle of M1: (0) absent or (1) present.
89. Protostylid of p4: (0) absent or (1) present.
90. Hypostylid of p4: (0) absent or (1) present.
91. Separation between protoconid and metaconid in p4: (0) absent or (1) present [enamel pit of Korth 1997]
92. Basioccipital: (0) little or not narrowed or (1) greatly [more than half] narrowed.
93. Enamel of cheek teeth: (0) continuous, (1) reduced to expose partial dentine tracts, or (2) reduced to expose full dentine tracts. [ordered]
94. Teeth: (0) rooted, (1) bearing reduced roots, or (2) not rooted. [ordered]
95. Main body of palate: (0) not expanded or (1) extends posteriorly to M3.
96. Lingual end of M1: (0) left open lingually without wear or (1) closed by lingual cingulum.
